# Opportunities and challenges to delivering a trial for depressive symptoms in primary care during the COVID-19 pandemic: insights from the Alpha-Stim-D randomised controlled trial

**DOI:** 10.1186/s13063-025-08772-3

**Published:** 2025-02-20

**Authors:** Shireen Patel, Priya Patel, Clement Boutry, Boliang Guo, Deborah Butler, Fred Higton, Rebecca McNaughton, Paul M. Briley, Christopher Griffiths, Neil Nixon, Vibhore Prasad, Kapil Sayal, David Smart, Azhar Zafar, Joe Kai, Richard Morriss

**Affiliations:** 1 NIHR ARC East Midlands, University of Nottingham, Nottingham, England; 2 School of Medicine, University of Nottingham, Nottingham, England; 3 Northamptonshire Healthcare NHS Foundation Trust, Northamptonshire, England; 4 Department of Population Health Sciences, King’s College London, London, England; 5 NHS Nottingham & Nottinghamshire, Nottingham, England; 6 The University of Northampton, Northampton, England; 7 Buckingham Medical School, University of Buckingham, Buckingham, England; 8 Leicester Diabetes Centre, Leicester General Hospital, Leicester, England; 9 NIHR School for Primary Care Research, University of Nottingham, Nottingham, England; 10 NIHR Mindtech Healthtech Research Centre (HRC), University of Nottingham, Nottingham, England; 11 NIHR Nottingham Biomedical Research Centre, University of Nottingham, Nottingham, England; 12 Institute of Mental Health, University of Nottingham, Triumph Road, Nottingham, NG7 2TU England

**Keywords:** Recruitment, Retention, RCT, DHIs, Depression, Alpha-Stim AID

## Abstract

**Background:**

Randomised controlled trials (RCTs) are widely regarded as the most powerful research design for evidence-based practice. However, recruiting to RCTs can be challenging resulting in heightened costs and delays in research completion and implementation. Enabling successful recruitment is crucial in mental health research. Despite the increase in the use of remote recruitment strategies and digital health interventions, there is limited evidence on methods to improve recruitment to remotely delivered mental health trials. The paper outlines practical examples and recommendations on how to successfully recruit participants to remotely delivered mental health trials.

**Methods:**

The Alpha Stim-D Trial was a multi-centre double-blind randomised controlled trial, for people aged 16 years upwards, addressing depressive symptoms in primary care. Despite a 6-month delay in beginning recruitment due to the COVID-19 pandemic, the trial met the recruitment target within the timeframe and achieved high retention rates. Several strategies were implemented to improve recruitment; some of these were adapted in response to the COVID-19 pandemic. This included adapting the original in-person recruitment strategies. Subsequently, systematic recruitment using postal invitations from criteria-specific search of the sites’ electronic health records was added to opportunistic recruitment to increase referrals in response to sub-target recruitment whilst also reducing the burden on referring sites. Throughout the recruitment process, the research team collaborated with key stakeholders, such as primary care clinicians and the project’s Patient and Public Involvement and Engagement (PPI/E) representatives, who gave advice on recruitment strategies. Furthermore, the study researchers played a key role in communicating with participants and building rapport from study introduction to data collection.

**Conclusions:**

Our findings suggest that trial processes can influence recruitment; therefore, consideration and a regular review of the recruitment figures and strategies is important. Recruitment of participants can be maximised by utilising remote approaches, which reduce the burden and amount of time required by referring sites and allow the research team to reach more participants whilst providing participants and researchers with more flexibility. Effectively communicating and working collaboratively with key stakeholders throughout the trial process, as well as building rapport with participants, may also improve recruitment rates.

## Background

Depression is the second leading cause of disability worldwide affecting 13% of the general population [1]. In the UK, the main treatments for depression are antidepressant medication and cognitive behavioural therapy (CBT). Whilst these treatments can be effective, 50% of people treated with a single antidepressant experiencing an inadequate response [2] and only 40% of those offered CBT attend two or more sessions, with 49% not progressing or recovering (National Health Service, 2020). In addition, patients who are referred for psychological therapies typically wait several weeks or months before gaining access to treatment [3] during which time mental health may deteriorate [4], highlighting the need for alternative evidence-based treatments that could be offered as part of primary care.

Randomised controlled trials (RCTs) are widely regarded as the gold standard for testing effectiveness [5]. However, recruitment to RCTs is often challenging [6]. Recruitment difficulties worsened during the COVID-19 pandemic with many trials struggling to recruit or ceasing recruitment [7, 8].

Poor recruitment of research participants is common in primary care research [9–12]. A review of 34 UK primary care RCTs found that only one-third met their recruitment timelines [13]. Delays in recruitment can increase costs, slow access to new treatments and hinder the assessment of effectiveness [14, 15]. According to a review [16], recruitment challenges occur at organisational, professional and patient levels. The review highlighted that improving recruitment requires strategies addressing all levels, particularly the professional level, since clinicians are crucial in providing patient access to RCTs [16].

Recruitment and retention to mental health RCTs is challenging [17] and is under-researched. To date, there have only been two reviews that have specifically investigated factors affecting recruitment into mental health RCTs [18, 19]. A systematic review investigating factors affecting the recruitment of participants into depression RCTs found that the decision to participate is significantly influenced by weighing the risks and rewards of RCT participation. Key factors included the service user’s current health condition when approached for participation, service providers’ and service users’ perspectives on the research and RCT interventions and the quality of interactions and relationships between service providers and service users These elements collectively impact the likelihood of participation in depression RCTs [18].

There has been a rise in remotely delivered or digital health interventions (DHIs) in research, with a recent shift towards online recruitment strategies, targeting populations not usually accessing mental health services.

A recent paper [20] explored perspectives on online and offline recruitment in mental health, identifying advantages such as improved accessibility. However, concerns about privacy and security, demographic preferences and cultural differences were identified as challenges. A meta-synthesis exploring factors impacting acceptability of DHIs found that addressing service users’ initial expectations of DHIs and the addition of rapid, responsive personal/human support, albeit offered remotely, could improve participant engagement with DHIs [21]. The literature on digital recruitment suggests that a combination of recruitment methods is more likely to optimise RCT recruitment and retention, acknowledging the need for further research on effective strategies.

Currently, there is limited evidence on the strategies or tools that could improve recruitment and retention in RCTs, especially those using primarily digital approaches in mental health research. Thus, it is crucial to report successful recruitment and retention methods and examine the real-life challenges of recruiting and retaining participants in primary care for studies on depressive symptoms. Additionally, understanding how contextual changes within study settings impact recruitment is essential.

## Aims

The Alpha-Stim-D trial was the first multi-centre RCT in England focused on addressing depressive symptoms in primary care [22]. In this paper, we present the Alpha-Stim-D trial research team’s experiences of conducting the trial during the COVID-19 pandemic. The COVID-19 pandemic had a significant impact on clinical and public health care research, COVID-19-related research was prioritised, and there was an NIHR statement to consider pausing or stopping non-COVID research or adopting different approaches to trial delivery which reduced the burden on sites and considered COVID-19 restrictions. When paused non-COVID-19-related research restarted, it was considerably impeded by lockdowns and social distancing policies, as well as reduced staffing capacity amongst clinical and non-clinical researchers and staff being furloughed. Face-to-face contact was often ceased, and research-related activities were adapted to become remote. Despite COVID-19 restrictions being lifted, remote delivery of RCTs continues to now be widely used.

The primary aim of the paper is to provide a detailed description of methods that facilitated the successful delivery of the RCT despite an initial 6-month delay. The paper reflects on the lessons learned and the methods utilised to overcome recruitment challenges to achieve recruitment and retention targets within the timeframe set prior to the COVID-19 pandemic. The paper concludes with recommendations on how participant recruitment and retention to RCTs might be improved.

## Methods

### Alpha-Stim-D trial study design and participants

The Alpha-Stim-D trial was a large multi-centre double-blind randomised controlled trial evaluating the clinical and cost effectiveness of the Alpha-Stim AID cranial electrical stimulation (CES) device for patients with depressive symptoms in primary care. The design and methods are outlined in more detail in a published trial protocol paper [22] and trial findings [23].

### Recruitment and retention strategies

Ethical approval was granted in February 2020 from the East Midlands—Leicester South Research Ethics Committee (REC Reference: 20/EM/0061). A month prior to the first UK COVID-19 lockdown, GP surgeries reported that there would need to be a minimum 6-month recruitment delay because they were under extreme pressures. Subsequently, the monthly recruitment rate had to increase from 10–12 participants to 13–14 participants to meet the recruitment target on time.

**Fig. 1:**
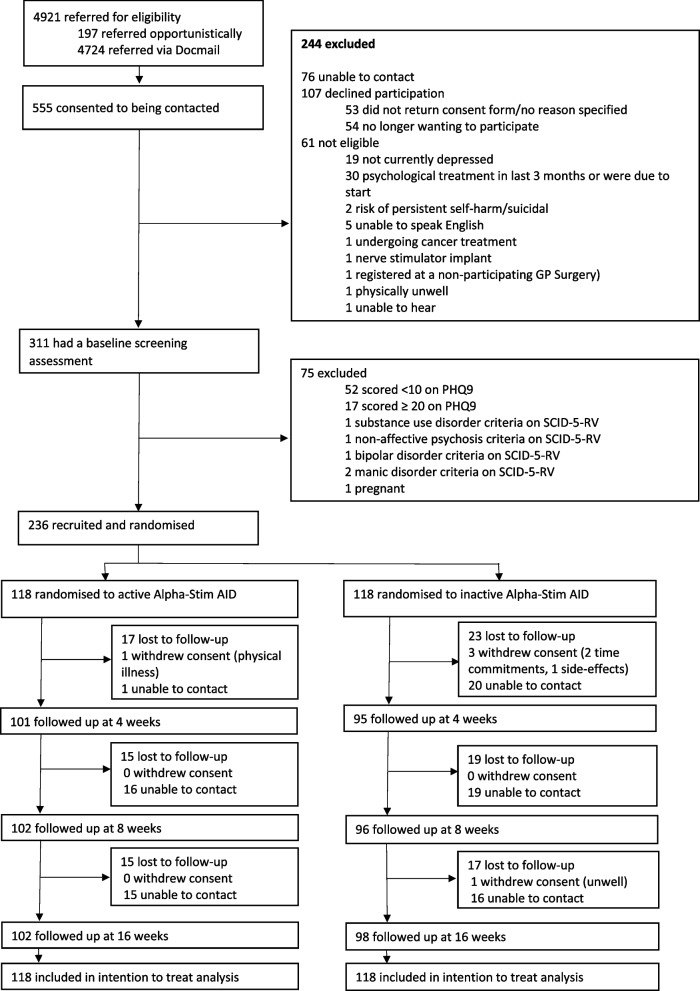
Alpha-Stim D trial participant flow

A diagram of participant flow to the Alpha-Stim-D trial is shown in Fig. 1.

A total of 4921 invites were sent between September 2020 and November 2022 (27 months) and 555 (11.3%) responded. Of these, 61 (11.0%) were not eligible and 183 (33.0%) declined to participate or were not able to be contacted. A further 75 (13.5%) were excluded during completion of the baseline assessment. In total, 236 participants (42.5%) were recruited to the trial, slightly exceeding the recruitment target of 230. An additional six participants were recruited because their baseline assessments had been pre-booked before the recruitment target had been met. We also maintained a high retention rate of 84% at 16-week follow-up; this was almost 10% above an anticipated follow-up rate of 75%. This led to a larger pool for analysis (199) than was required in the sample size calculation.

**Fig. 2:**
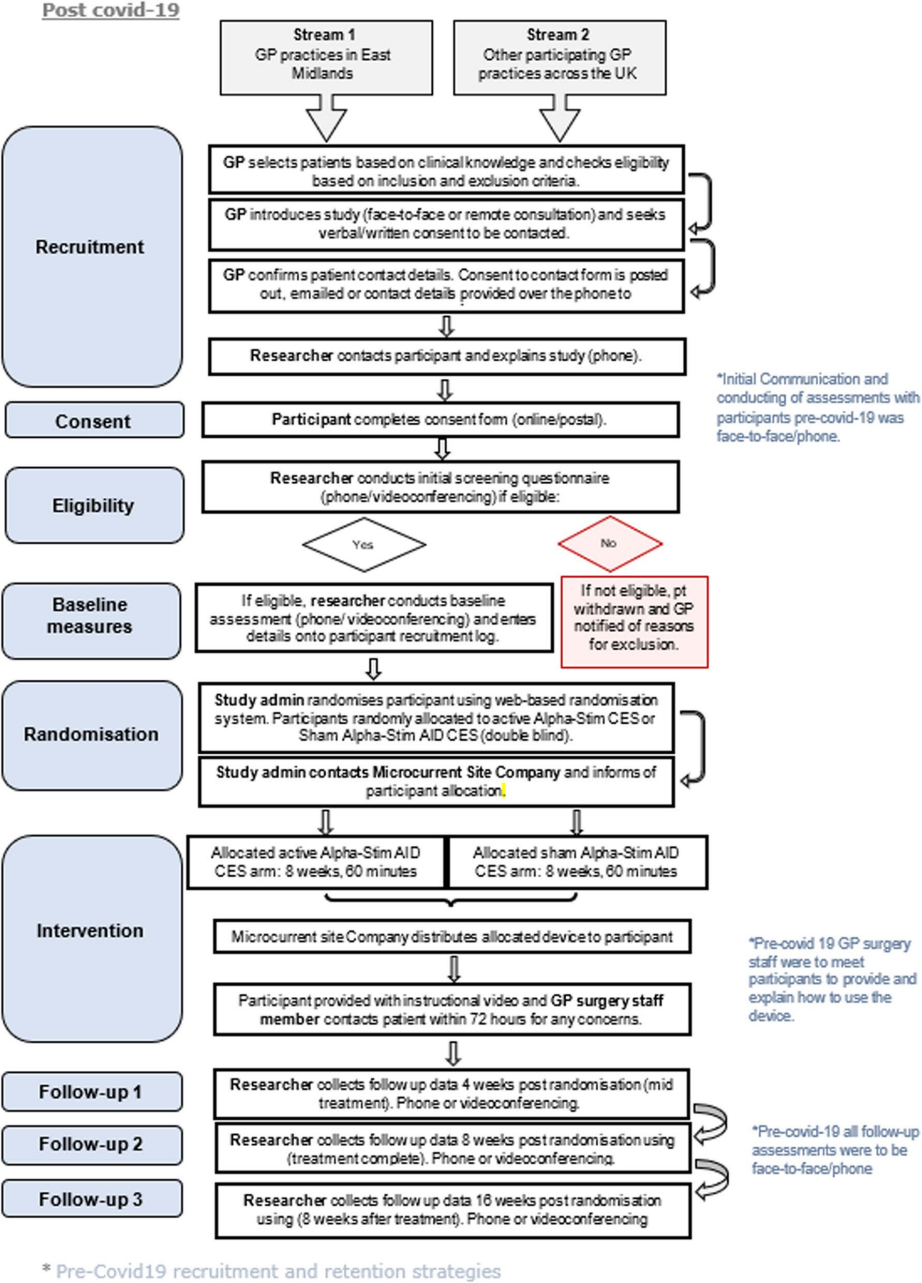
Modified recruitment process

Figure 2 illustrates the modified recruitment process to facilitate recruitment because of COVID-19 restrictions.

Table 1 provides a breakdown of target versus actual participant recruitment throughout the trial. Although the trial recruited to time and target, the initial recruitment plan was adapted to the evolving situation of the COVID-19 pandemic. Following the initial 6-month delay to recruitment, the first modification was made to introduce remote recruitment approaches (strategy 1).

**Table 1:** Revised target versus actual monthly recruitment figures

Month	Month	Monthly target recruitment	Monthly actual recruitment	Total recruitment	Recruitment rate (%)
1	September 2020	14	6	6	2.6
2	October 2020	14	4	10	4.3
3	November 2020	14	2	12	5.2
4	December 2020	14	7	19	8.3
5	January 2021	14	16	35	15.2
6	February 2021	14	17	52	22.6
7	March 2021	14	17	69	30
8	April 2021	14	8	77	33.4
9	May 2021	14	13	90	39.1
10	June 2021	13	17	107	46.5
11	July 2021	13	11	118	51.3
12	August 2021	13	13	131	57.0
13	September 2021	13	20	151	65.7
14	October 2021	13	11	162	70.4
15	November 2021	13	26	188	81.7
16	December 2021	13	37	225	97.8
17	January 2022	13	11	236	102.6

### Strategy 1: remote recruitment processes (September 2020–January 2022)

Following the government imposing a national lockdown in March 2020 due to COVID-19 pandemic, a substantial amendment was submitted to the Health Research Authority (HRA) Research Ethics Committee (REC). Changes made to the recruitment approach consisted of offering assessments remotely—over the telephone or via Microsoft Teams to minimise face-to-face contact and overcome COVID-19 restrictions. The added benefit of remote completion of assessments was that it provided greater flexibility for participants and study researchers and reduced logistical barriers. Initial training on the use of the device was anticipated to be at the GP surgery by a healthcare professional. To overcome this, an instructional training video was developed in collaboration with a Patient and Public Involvement and Engagement (PPI/E) representative, which provided instructions on the use of the device and offered tips whilst showing participants how to log device usage. This enabled devices to be directly posted to participant’s homes and for participants to be able to view the instructional video on YouTube (https://youtu.be/5eti64s0hys). The research team arranged for devices to be sent directly to participants home by the distributers along with pre-paid envelopes so that they could return the devices with no additional cost incurred to them.

Table 1 shows that, in the first few months of recruiting using opportunistic methods at primary care consultations, recruitment rates were under target with 37 fewer participants than required recruited by month 4, which is not uncommon in RCTs [24]. Recruitment figures then increased steadily and 90 participants had been recruited by month 9. Eleven months into the trial (end of July 2021), with only 6 months left to recruit, 118 participants (just over 50%) had been recruited, indicating a need for further efforts to improve recruitment. Strategy 2 was then introduced.

### Strategy 2: utilising multiple referral approaches (May 2021–November 2022)

During the initial set-up stage of the trial, GPs suggested that an opportunistic approach whereby potential participants were approached about the study, based on clinical knowledge during consultation, would be most feasible. This approach was perceived to be straightforward and require minimal input from GPs, as they only had to obtain verbal consent to being contacted from participants and share contact details with the study researchers.

As shown in Table 1, 6 months into the trial study researchers recognised there was a need for additional methodological changes to be implemented to increase referral rates, particularly as fewer face-to-face mental health consultations were taking place. Therefore, systematic recruitment as a referral approach was introduced. This referral approach consisted of a search being conducted by GP surgeries of patients’ primary care medical records to identify potentially eligible participants. A letter of invitation, participant information sheet and a pre-paid envelope were then sent from the GP surgery. Interested patients were asked to complete an expression of interest form and return this to the research team.

**Fig. 3:**
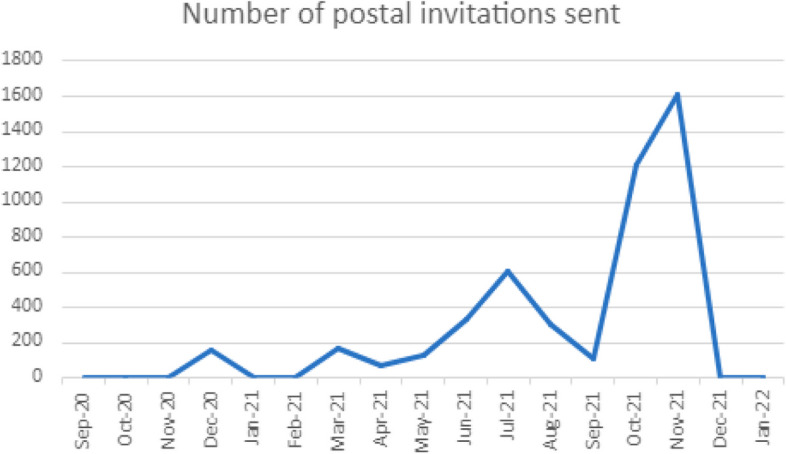
Number of postal invitations sent out

Figure 3 shows the number of postal invitations sent out by GP surgeries; there was an increase between May 2021 and July 2021 and again from September 2021 which peaked during November 2021 (1600 postal invites were sent in November). This increase in referrals translated into the largest number of participants being recruited in November and December 2021 (26 and 37 participants respectively) as shown in Table 1. This highlights the effectiveness of the postal invitation referral approach in comparison to the opportunistic approach that was primarily relied upon prior to May 2021.

We believe that in addition to the recruitment methodological changes utilised due to COVID-19 pandemic, the following additional strategies (3 and 4) contributed to successful recruitment and retention of clinicians and participants.

### Strategy 3: collaborative working from study inception to dissemination (November 2019–January 2023)

An integral aspect that led to successful recruitment of the Alpha-Stim-D trial was collaborative working, which involved GP surgeries, Clinical Research Networks (CRNs) and Patient and Public Involvement and Engagement (PPI/E) representatives.

#### GP surgeries

The study team recognised the importance of setting up initial meetings with staff at GP surgeries to introduce the trial. Due to the COVID-19 pandemic, all meetings with recruiting sites took place over Microsoft Teams. Study researchers highlighted the benefits of taking part in the research and clarified the trial processes. GP surgeries were also informed that in line with recommended NHS service support for research they would receive £94.92 for each participant recruited; this was to reimburse primary care staff for their contribution and time. Where possible, a member of the GP practice team was designated as the main point of contact who would liaise with the lead researcher with referrals and any queries. Trial processes were kept simple and clear for primary care staff to keep their workload to a minimum.

We regularly updated GP surgeries in terms of recruitment figures and sought feedback on any recruitment challenges experienced. We sent out newsletters to all participating sites updating them on recruitment progress and commending top recruiting sites. Between November 2021 and January 2022, a total of 74 participants were recruited enabling us to meet our recruitment target 14 days ahead of time.

#### Clinical Research Network (CRN) support

The study was adopted onto the NIHR research portfolio and, as such, was eligible for CRN support. However, between April 2020 and August 2020, CRN support was restricted to COVID-19 studies only. This meant that CRNs could not support the trial in terms of releasing their staff to support the trial with the promotion or recruitment of participants. The CRN in the East Midlands commenced promoting and supporting the trial in August 2020. In January 2021, the CRN in the Thames Valley and South Midlands region also commenced promoting the study to GPs in their region. The study lead researcher arranged monthly meetings with the CRN leads, updates were provided, and CRN leads chased up GP surgeries if required. CRN researchers were provided with a half-day training on trial processes and the assessments. This enabled some CRN researchers to provide additional support to the study team by conducting participant assessments.

#### Working in collaboration with PPI/E representatives

PPI/E input was at all stages, from proposal to dissemination. During set up, three PPI/E representatives were recruited as permanent members of the study team. The PPI/E representatives provided input on all patient-facing documents. We collaborated with PPI/E representatives on the development of the instructional YouTube video and study researchers continued to hold regular remote meetings with PPI/E representatives. This enabled researchers to update PPI/E representatives and to share ideas and help overcome any recruitment barriers. This was also highlighted by our PPI/E representatives to be beneficial for their mental health, particularly during the pandemic. Our PPI/E representatives played a pivotal role in the dissemination of findings and interpretation of results, as well as sharing their experiences of being involved in the trial through national conferences and online video testimonials.

#### Leadership, peer support and provision of training

The study was guided by strong leadership, which included regularly monitoring recruitment rates, engaging with researchers and GP surgery staff and evaluating the timing and application of recruitment strategies, such as opening additional referral sites. This was critical to achieving recruitment and retention goals. Although all study researchers were required to work remotely, they provided regular support to one another. Prior to any researcher completing assessments, the lead researcher provided training. Throughout the trial, the lead researcher arranged weekly meetings with trial researchers to discuss participant recruitment and trial progress.

### Strategy 4: role of study researchers in communicating with participants and collecting data (September 2020–January 2022)

The study researchers comprised one full-time lead researcher, two part-time research assistants and a research nurse from the Clinical Research Network (CRN) who offered 1 day a week on the trial. Following referral by the primary care team, the research team made initial contact with participants to introduce the study, determine eligibility, consent participants and conducted baseline and follow-up assessments. We believe the following aspects may have contributed to improved recruitment and retention rates.

#### Introducing the study to participants

The initial communication from the study team with interested participants was in the form of a verbal conversation over the telephone. Study researchers informed participants about the study rationale, potential benefits and costs involved in taking part in the trial and what their participation would involve. Potential participants were offered the opportunity to ask questions and express any initial concerns, facilitating rapport building with the study researchers. The decision to communicate with participants verbally prior to emailing a copy of the Participation Information Sheet (PIS) was to reduce the likelihood that participants might be overwhelmed by a long-written document.

#### Communication skills of the researchers

All researchers were approachable, trustworthy and had good knowledge of the trial. They individualised their communication style to the needs and values of the participants. For example, completion of follow-up assessments with a study researcher was offered over the phone or via video-calling based on participant preference. Study researchers dedicated additional time to speak to the participants and listen to their experiences. A qualitative study embedded within the Alpha-Stim D trial was conducted to explore participant experience of using the Alpha-Stim AID device. The findings have been published [25].

Following completion of all follow-up assessments, participants were emailed Amazon gift vouchers, which they were able to use online, as a token of appreciation. Upon completion of the trial and data analysis, all participants were emailed a summary of the findings. This was in the form of a lay person two-page summary of the key study findings and its implications. The summary was produced in collaboration with PPI/E representatives to ensure that it was appropriate and understandable.

In addition, study researchers also communicated to GPs any risk issues that were highlighted at baseline or follow-up assessments. This ensured that participants were followed up by their clinicians and risk managed.

#### Continuity and flexibility of study researchers

Outcome measures were intended to be simple and concise requiring minimal time commitment from participants. These were completed with the researcher over the phone or via Microsoft Teams depending on patient preference. Where possible, the same researcher conducted the baseline and follow-up assessments to ensure continuity and consistency. All participants were contacted via text message a week prior to their follow-up assessments to arrange a time for this. If participants did not respond, two reminder texts were sent out followed by a phone call if no response was received.

Researchers were flexible, offering assessments outside of their working hours to accommodate participants’ lifestyles where possible. This included assessments in the evenings and occasional weekend working. This both facilitated recruitment and retention rates.

**Fig. 4:**
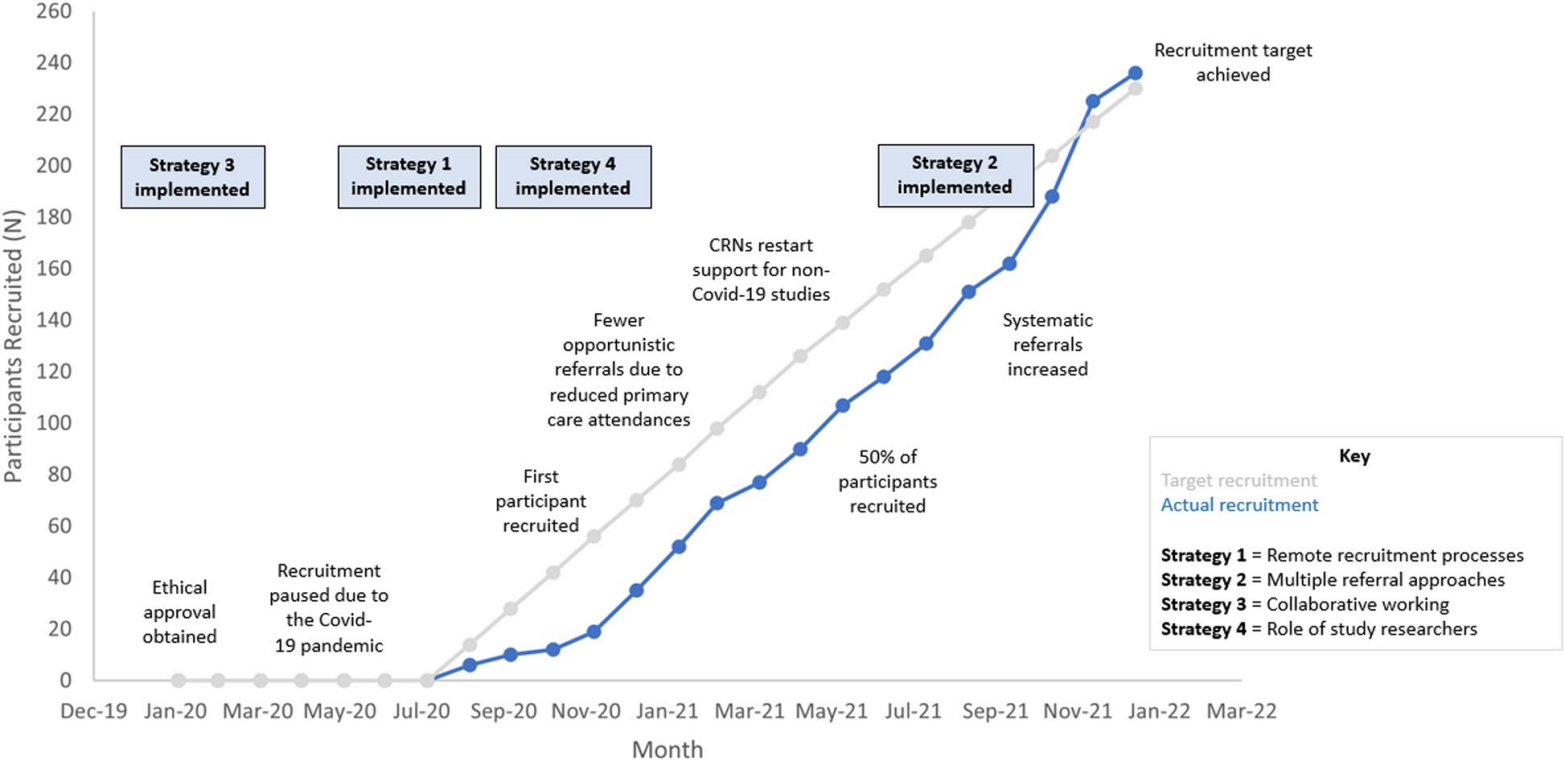
Timeline of recruitment

Figure 4 illustrates a timeline of recruitment, the onset of strategies and milestones.

A summary of the strategies utilised to improve recruitment figures and perceived impacts are presented in Table 2.

**Table 2:** Strategies utilised to boost recruitment rates

Strategy	Impact
1: Remote recruitment processes • Assessments via telephone call or Microsoft Teams • Device sent via post rather than collected from GP surgery • YouTube instructional video rather than GP surgery staff showing participants how to use device	The trial was able to recruit safely and effectively throughout COVID-19 pandemic. It also improved reach as participants could be recruited from a wider geographical area and increased efficiency due to reduced travel time The instructional video reduced GP staff workload and facilitated recruitment during the COVID-19 pandemic
2: Utilising multiple recruitment approaches • Opportunistic • Systematic	Increased referral numbers and provided GPs with multiple referral options
3: Collaborative working from study inception to dissemination • GP surgeries and academic GPs • Working with primary care CRN teams • PPI/E representatives • Peer support and provision of training	Ensured that the trial was feasible and acceptable for recruiting sites and trial participants The provision of training ensured that those involved in supporting recruitment felt supported and motivated to engage in the trial
4: Role of researchers in communicating with participants and collecting data • Introducing the study to participants • Communication skills of the researchers • Continuity and flexibility of study researchers	Participants were able to build a rapport with researchers which enabled them to feel comfortable and supported. This may have improved engagement with the trial and influenced retention rates

## Discussion

Delivering a trial for depressive symptoms in primary care during the COVID-19 pandemic presented both opportunities and challenges. This paper aimed to present the key lessons learned during recruitment into the Alpha-Stim-D multi-centred RCT. Despite the COVID-19 pandemic causing delays in recruitment, the trial successfully recruited 236 participants to time and target and achieved an overall retention rate of 84%. This paper highlights the initial challenges faced by the research team and summarises the strategies utilised to overcome recruitment barriers. The novelty of this work lies in its strategic adaptation to unprecedented conditions, the COVID-19 pandemic. This study not only maintained high recruitment and retention rates despite significant barriers but also demonstrated how remote and multifaceted recruitment strategies can broaden geographical reach, reduce logistical burdens and increase participant access. The insights gained are not just context-specific but propose scalable strategies for future digital RCTs, suggesting a paradigm shift in clinical trial recruitment and retention practices beyond the pandemic era.

### Implications for future studies

Key findings from the paper highlight that carefully adapting remote recruitment approaches enabled the study team to safely recruit participants and offer participants an intervention that could be used independently at home during a time when access to alternative forms of treatment for depressive disorders (e.g. face to face psychotherapy) was constrained due to social restrictions placed by the COVID-19 pandemic. We were able to increase our geographical reach by recruiting participants from across England and maximise efficiency as it removed travel for both participants and researchers. Given the increase in use and accessibility of remote recruitment strategies post COVID-19 pandemic [20], the recommendations offered in this paper are also relevant for RCTs conducted outside of a pandemic.

The findings also highlight the benefits of utilising multiple referral approaches. The COVID-19 pandemic reduced in-person mental health consultations with the GP [26]; therefore, the trial could not rely solely on opportunistic referrals. Using a combination of approaches boosted referral and recruitment rates. Researchers should be aware that, whilst postal invitations can be more costly, this approach can enable a larger number of potential participants to be contacted within a short period. It is also important that the letter is from a credible source, i.e. the GP surgery. Increasingly, this can be done via text messaging and link within the message as now routinely happens for other service contact/communications or requests from GP. The opportunistic approach also had its own advantages, with a greater percentage of eligible referrals as clinicians reviewed patient eligibility against recruitment criteria on a case-by-case basis. Therefore, applying a combination of referral approaches may provide a balance of volume and quality of referrals and may also reduce additional workload for clinicians which can often be a barrier to recruitment [16, 18]. It is worth acknowledging that SMS campaigns are becoming more common in primary care communication now. Primary care favours SMS campaigns because they are currently free (paid for by the NHS) whilst postal letters are more time-consuming to print, package and post. The effectiveness of SMS campaigns for recruitment and retention warrants further exploration.

Finally, the paper highlighted the key role research teams play in facilitating recruitment and retention. Consistent with a systematic review [22], participants and GPs both highlighted that they valued researcher support as it facilitated study understanding and motivation to continue with the trial. Postal recruitment conversion rates were lower than opportunistic recruitment highlighting that patients may have misunderstood the study or not felt that it was relevant to their symptoms. Within an opportunistic approach, clinicians could gauge patient understanding, allay any concerns and ensure that their patients comprehended study rationale and processes. Therefore, providing support to service providers and service users may be important to maintain engagement and adherence in research trials.

This paper highlights the strategies that could be utilised to improve recruitment and retention of participants to a remotely delivered RCT. A multifaceted approach that adapts to changing circumstances and feedback is more likely to succeed than one limited to one or two fixed strategies. Future digitally delivered RCTs could build on the reflections presented in this paper. Furthermore, there is a need to develop a theoretical framework to improve recruitment and retention to trials.

### Strengths and limitations

Whilst our findings reflect an RCT for depressive symptoms, the lessons learnt can be applied to other digital trials in primary care and secondary care because they provide potential strategies to implement when recruiting patients to any RCT.

It is worth noting that we did not interview clinicians or participants specifically about their views on the recruitment processes or the barriers and facilitators so we can only infer what successful recruitment and retention strategies might have been. Given the novelty of the intervention, a neuromodulation device that could be used at home, the trial may have generated more interest from GPs and people with depression than other more conventional medication and psychological interventions. Interviews with Alpha-Stim AID study participants indicate a desire to try an alternative to psychotherapy and antidepressants [25, 27]. The COVID-19 pandemic and its restrictions may have particularly encouraged the use of treatments that can be utilised at home when other forms of treatment such as face-to-face psychological treatment might have seemed less accessible. It is also important to recognise that in areas of greater digital deprivation the digital methods may be more unequal. This might explain the success of recruitment to the trial that might not be generalisable outside COVID.

It would have been valuable to try to pin down which were the most impactful strategies; however, it is not entirely possible to consider all the different research strategies in isolation and identify how one on its own made more of a difference than another. Some strategies around PPI/E involvement and the way the team worked were constant throughout. Therefore, it is important to recognise that there were a range of successful strategies that built on each other.

It is also worth highlighting that some of the recruitment approaches we propose may not be possible in other RCTs due to financial, regulatory or other constraints, such as issues around accessibility, obtaining consent or carrying out assessments that cannot be adapted for remote delivery. Not everyone has access to technology or the skills to use it, potentially leading to selection bias and inequalities. This may be particularly true for RCTs involving medicinal products, especially new or invasive treatments, or studies requiring specialised equipment or monitoring. Postal invitations incur postage costs and research budgets may restrict the number of postal invites that can be sent out. Though this can often be effectively negotiated using text messaging to patients’ phones by service sites with links to information. Few under-18-year-olds were recruited to the RCT, and they may not use or engage with traditional postal mail. Furthermore, flexible working patterns may not be appropriate for all researchers as they may have other priorities not allowing them to work outside of office hours. Completion of outcome assessments with researchers also requires additional researcher time and has cost implications.

Recommendations for researchers:

1. Involve PPI/E representatives in all aspects of the RCT. PPI/E is essential from the inception of trial through to dissemination.
2. Adapt recruitment approaches so that that RCT processes are simple and acceptable for clinicians and participants.
3. Where possible, utilise a combination of referral approaches to employ the advantages of each approach and maximise likelihood of meeting recruitment targets.
4. Offer regular meetings and provide written updates for everyone involved in the trial, including referring sites, participants and study collaborators.
5. Recognise the importance of the role of the study researchers. The way in which the RCT is communicated to referring sites and potential participants may play a key role in determining study participation. Provide support and supervision for the researchers.
6. Incorporate leadership and trial management meetings to monitor RCT progress and adapt strategies throughout the trial based not only on recruitment data but also on the feedback and “soft” intelligence regarding perceived barriers and facilitators to recruitment.

## Conclusions

The COVID-19 pandemic changed the way that research was conducted, encouraging remote recruitment approaches. Although the pandemic no longer hinders research activities, the lessons learnt can be applicable to digital trials delivered more widely. Overall, researchers recruiting to RCTs should consider how the research processes can influence recruitment. Our findings suggest that recruitment can be maximised by reducing the burden placed on referring sites and utilising remote approaches to reach participants from a wider range of geographical locations and offer more flexibility in when and where the assessments are carried out. However, the findings highlight that building rapport and maintaining regular communication with both participants and referring sites is key when utilising remote approaches. This helps to maintain both referral and participant retention rates. These insights will aid future researchers and stakeholders with an interest in the field. They will also promote transparency and motivate others to evaluate their recruitment strategies, fostering a more standardised and effective approach.

## Data Availability

Not applicable as no data were collected for this paper.
